# Quantitative Polymerase Chain Reaction Coupled With Sodium Dodecyl Sulfate and Propidium Monoazide for Detection of Viable *Streptococcus agalactiae* in Milk

**DOI:** 10.3389/fmicb.2019.00661

**Published:** 2019-03-29

**Authors:** Yankun Zhao, He Chen, Huimin Liu, Jianxing Cai, Lu Meng, Lei Dong, Nan Zheng, Jiaqi Wang, Cheng Wang

**Affiliations:** ^1^Institute of Quality Standard and Testing Technology for Agro-Products, Xinjiang Academy of Agricultural Sciences, Urumqi, China; ^2^Ministry of Agriculture and Rural Affairs-Laboratory of Quality and Safety Risk Assessment for Agro-Products, Urumqi, China; ^3^Key Laboratory of Agro-Products Quality and Safety of Xinjiang, Urumqi, China; ^4^Ministry of Agriculture Laboratory of Quality and Safety Risk Assessment for Dairy Products (Beijing), Institute of Animal Science, Chinese Academy of Agricultural Sciences, Beijing, China

**Keywords:** *Streptococcus agalactiae*, propidium monoazide, sodium dodecyl sulfate, qPCR, milk

## Abstract

*Streptococcus agalactiae* is an important pathogen causing bovine mastitis. The aim of this study was to develop a simple and specific method for direct detection of *S. agalactiae* from milk products. Propidium monoazide (PMA) and sodium dodecyl sulfate (SDS) were utilized to eliminate the interference of dead and injured cells in qPCR. Lysozyme (LYZ) was adopted to increase the extraction efficiency of target bacteria DNA in milk matrix. The specific primers were designed based on *cfb* gene of *S. agalactiae* for qPCR. The inclusivity and exclusivity of the assay were evaluated using 30 strains. The method was further determined by the detection of *S. agalactiae* in spiked milk. Results showed significant differences between the SDS–PMA–qPCR, PMA–qPCR and qPCR when a final concentration of 10 mg/ml (*R*^2^ = 0.9996, *E* = 95%) of LYZ was added in DNA extraction. Viable *S. agalactiae* was effectively detected when SDS and PMA concentrations were 20 μg/ml and 10 μM, respectively, and it was specific and more sensitive than qPCR and PMA–qPCR. Moreover, the SDS–PMA–qPCR assay coupled with LYZ was used to detect viable *S. agalactiae* in spiked milk, with a limit of detection of 3 × 10^3^ cfu/ml. Therefore, the SDS–PMA–qPCR assay had excellent sensitivity and specificity for detection of viable *S. agalactiae* in milk.

## Introduction

*Streptococcus agalactiae* is a Gram-positive, pathogenic bacterium that commonly cause subclinical mastitis in dairy cattle (Santos et al., 2013). It is the most infectious and the fastest spreading pathogen among *Streptococcus* spp., and also an important infectious pathogen for humans and animals with potential risk for food poisoning of salad, cheese, milk, fish, and meat (Pimentel et al., 2016; Kaczorek et al., 2017). Microbiological culture methods are routinely used to identify cows infected with *S. agalactiae.* Although cultivation of specimens and emergence of bacterial colonies is still the gold standard to detect *S. agalactiae*, it is laborious and time consuming and has low specificity (Gholamreza et al., 2015). Therefore, establishing a rapid, accurate and sensitive detection of *S. agalactiae* has significance for public health.

Currently various rapid detection methods, such as PCR, quantitative PCR (qPCR), real-time fluorescence quantitative (real-time PCR), and multiplex quantitative PCR (M-qPCR) are regarded as reliable to detect *S. agalactiae* in milk or other food products (Pai et al., 2000; Ayman et al., 2015; Sebastião et al., 2015; Bosward et al., 2016). However, the RNA of the dead bacteria cells will degrade gradually and is no longer produced, and so RT-PCR based on the level of RNA can determine the presence or absence of bacteria with strong activity (Graiver et al., 2010; Kim et al., 2011), but there are some doubts about this technology at present. Therefore, using common PCR it is enormously difficult to distinguish between viable and dead bacterial cells (Varma et al., 2009). In addition, the naked DNA can persist in the environment long after cell death, qPCR, and M-qPCR generate high false-positive rates due to DNA persistence after cell death and therefore overestimate infection risks (Niu et al., 2018). To overcome this limitation, DNA-intercalating dyes such as propidium monoazide (PMA) are a promising way to selectively detect viable cells. Viable cells have a complete cell membrane structure, which can exclude PMA, but PMA can penetrate cells that are dead or have a damaged membrane. The PMA forms covalent bonds with DNA under intense light, PCR amplification of such modified DNA is strongly inhibited, allowing unbound DNA from viable cells to be amplified and detected in subsequent PCR (Nocker et al., 2006, 2009).

However, PMA cannot completely penetrate the dead cell membrane, resulting in false-positives (Lee and Levin, 2009) – treatment of cells with sodium dodecyl sulfate (SDS) along with PMA can solve this practical problem (Takahashi et al., 2017). At present, there is no report on the detection of viable *S. agalactiae* under a background of thermal injury using SDS–PMA–qPCR technology.

The aim of the present work was to establish a novel qPCR assay coupled with SDS and PMA to detect viable *S. agalactiae* cells in milk. Moreover, optimization of lysozyme (LYZ), SDS, and PMA was designed to improve the detection by SDS–PMA–qPCR of viable *S. agalactiae* cells.

## Materials and Methods

### Bacterial Strains and Culture Conditions

The *S. agalactiae* (ATCC12386) was cultured overnight in blood agar plates (Beijing Land Bridge Technology Ltd., Beijing, China). A single colony was transferred to 10 ml of brain heart infusion (BHI, Beijing Land Bridge Technology Ltd.) medium, and incubated in a rotary shaker (180 rpm) at 37°C for 3 h. The suspension was then centrifuged at 10,000× *g* for 3 min at 4°C to harvest cells. The letter was resuspended in sodium chloride solution (0.85%, Beijing Land Bridge Technology Ltd.) to obtain concentrations ranging from 10^1^ to 10^8^ cfu/ml. Aliquots (100 μl) of the serial dilutions were spread onto BHI agar plates. Viable cell numbers were determined by counting colonies after plates were incubated at 37°C for 24 h.

### Preparation of Dead Cells

To obtain dead cells, a centrifuge tube containing 500 μl of cell suspensions (8 × 10^7^ cfu/ml) was heated at 90°C in a water bath for 20 min. Plate counting confirmed that no bacteria had survived in suspensions.

### Genomic DNA Extraction

The bacteria pellet was first resuspended in TE buffer (10 mM Tris–HCl and 1 mM EDTA, pH 8.0), and added to the LYZ stock solution of optimal concentration. The mixture was incubated at 37°C for 1 h.

The DNA templates were extracted using the cetyl trimethyl ammonium bromide (CTAB) method by modifying the protocol of Zhou et al. (1996) and Kachiprath et al. (2018). DNA extraction was performed with 1 ml of 2% CTAB [100 nM Tris–HCl, (pH 8.0), 1.4 M NaCl, 20 nM EDTA], and 200 mg of micro glass beads; the mixture was ground at 1800 rpm for 120 s in a TL2020 high-throughput tissue homogenizer (0401261, DHSBIO), sediment samples were suspended in 20 μl of Proteinase K (20 mg/ml) and the suspension was incubated at 55°C for 30 min and 70°C for 20 min. Genomic DNA in resulting lysate was centrifuged at 14,000× *g* for 5 min and the supernatant was purified by extraction with an equal volume of phenol: chloroform:iso-amyl alcohol (25:24:1, vol/vol) and the step repeated. The aqueous phase was precipitated with 560 μl of iso-propanol and the crude nucleic acid from the aqueous phase was pelletized by centrifugation at 4°C, 14,000× *g* for 15 min, and the supernatant was discarded. The DNA pellet washed with 1 ml of ice-cold 70% (vol/vol) ethanol, and then it followed by absolute ethanol was air-dried and resuspended in TE buffer. The DNA concentration and quality were determined with a Nanodrop 1000 spectrophotometer (Thermo Fisher Scientific, Waltham, MA, United States). The extracted genomic DNA samples were stored at −20°C until use.

### Inclusivity and Exclusivity of Primer Tests

Inclusivity and exclusivity tests for the primers were performed using a panel of 30 strains, including 20 *S. agalactiae* and other common pathogenic bacterial species in raw milk (Table 1). The primers 5′-ATGGGATTTGGGATAACTAAGCTAG-3′ (forward) and 5′-AGCGTTATTCCAGATTTCCTTAT-3′ (reverse) targeted the specific *cfb* gene of *S. agalactiae* (Kaczorek et al., 2017). The primers were synthesized by Shanghai Sangon Biotech Co., Ltd. (Shanghai, China). All real-time qPCR was performed in a 96-well microtiter plate and amplification detections were performed in CFX96 real-time PCR systems (Bio-Rad, CA, United States). Reactions were performed in a 25-μl system containing KAPA PROBE FAST qPCR Master Mix (Sigma-Aldrich, St. Louis, MO, United States), 10 ng/μl of sample templates, 0.4 μM of each of the primers and 8.5 μl of distilled H_2_O. The cycling protocol included an initial 2-min denaturation step at 94°C followed by 30 cycles of repeated denaturation at 94°C for 30 s and annealing and extension at 50°C for 1 min (Kaczorek et al., 2017). Fluorescent data were acquired during the annealing and extension phase. A negative control with water was included in each qPCR reaction. After amplification, qPCR products were subjected to 1.5% agarose gel electrophoresis and visualized with a UV transilluminator (Bio-Rad, Hercules, CA, United States) after staining with nucleic acid dye (Qiagen, Hilden, Germany).

**Table 1:** Bacteria strains used for specificity test of primers.

Bacterial species	Strain ID	Source^1^	PCR result^2^	Origin/host
*Streptococcus agalactiae*	ATCC12386	ATCC	+	/
	XH07	XJAAS	+	Milk of bovine mastitis
	XH07-2	XJAAS	+	Milk of bovine mastitis
	XH12	XJAAS	+	Milk of bovine mastitis
	XH33	XJAAS	+	Milk of bovine mastitis
	XH45	XJAAS	+	Milk of bovine mastitis
	XH46-1	XJAAS	+	Milk of bovine mastitis
	XH14-2	XJAAS	+	Milk of bovine mastitis
	XLS008	XJAAS	+	Milk of bovine mastitis
	XL48-2	XJAAS	+	Milk of bovine mastitis
	XL30-2	XJAAS	+	Milk of bovine mastitis
	XL13-1	XJAAS	+	Milk of bovine mastitis
	XL13-2	XJAAS	+	Milk of bovine mastitis
	XL44	XJAAS	+	Milk of bovine mastitis
	XM72-4	XJAAS	+	Milk of bovine mastitis
	XM34-3	XJAAS	+	Milk of bovine mastitis
	XM25	XJAAS	+	Milk of bovine mastitis
	XB16	XJAAS	+	Milk of bovine mastitis
	XD9-2	XJAAS	+	Milk of bovine mastitis
	XD50-1	XJAAS	+	Milk of bovine mastitis
*Cronobacter sakazakii*	CICC21640	CICC	−	/
*Bacillus cereus*	ATCC11778	ATCC	−	/
*Escherichia coli*	ATCC25922	ATCC	−	/
*Shigella flexneri*	ATCC12022	ATCC	−	/
*Cronobacter muytjensii*	ATCC51329	ATCC	−	/
*Salmonella enterica*	ATCC14028	ATCC	−	/
*Lactobacillus plantarum*	ATCC8014	ATCC	−	/
*Enterococcus faecalis*	ATCC29212	ATCC	−	/
*Staphylococcus aureus*	ATCC6538	ATCC	−	/
*Streptococcus salivarius*	ATCC14485	ATCC	−	/

*^1^CICC, China Center of Industry Culture Collection (Beijing, China); CMCC, China Medical Culture Collection (Beijing, China); ATCC, America Type Culture Collection (Rockville, MD, United States); XJAAS, Xinjiang Academy of Agriculture science (Xinjiang, China). ^2^+ = positive results; − = negative results. ^3^/ = purchased standard strain.*

### Optimization of SDS Treatment

The SDS (Wako Pure Chemical Industries, Osaka, Japan) was dissolved in distilled water to obtain a 10^4^ μg/ml stock solution and then sterilized by autoclaving for 20 min at 121°C. The SDS treatment was as described by Dong et al. (2018). The SDS solutions were prepared with final respective concentrations of 0, 5, 10, 15, 10, 25, 40, 50, 100, 150, 200, 250, and 500 μg/ml. After 6 h of incubation in BHI medium, *S. agalactiae* suspensions were centrifuged at 10,000 *g* for 10 min at 4°C. The pellets were then resuspended in 0.1% (wt/vol) peptone water with serially diluted SDS. Blood agar plates were used to enumerate the viability of surviving cells in suspension. Of cell suspension of different concentrations, 100 μl was plated on blood agar plates at 37°C for 24 h. The numbers of viable cells were confirmed from bacterial counts. The optimized concentration of SDS was determined by maximizing the SDS concentration inhibiting the amplification of dead *S. agalactiae* and enhancing PMA absorption without sacrificing viable cells.

### Validation of LYZ Concentrations

To determine the optimum LYZ concentration, four aliquots of viable *S. agalactiae* were obtained also as described above suspension (10^7^ cfu/mL) were respectively treated with different LYZ final concentrations: 5, 10, 15, and 20 mg/ml. The optimal concentration of LYZ was determined using a good linear standard curve based on qPCR results.

### Optimization of PMA Treatment

The PMA (Biotium Inc., Hayward, CA, United States) was dissolved in 20% dimethyl sulfoxide (Sigma-Aldrich) to produce a 10 mM stock solution, and then stored at −20°C in darkness until use. The tested *S. agalactiae* mixtures were prepared by mixing 1 ml of viable cells with 1 ml of dead cells. Each 2-ml aliquot of the prepared bacterial suspension was treated with the optimized SDS concentration, and treated with different PMA final concentrations of 0, 10, 20, 30, 40, and 50 μM in Eppendorf tubes and incubated in darkness for 20 min. Then the tubes, with lids removed, were exposed to a 500-W halogen light source at a 20-cm distance for 10 min while placed in an ice bath. The samples were centrifuged at 10,000 *g* for 10 min and washed twice with PBS before DNA extraction. According to the threshold cycle (Cq), the minimal PMA concentration was accepted as the optimal PMA condition.

### Sensitivity of SDS–PMA–qPCR

The sensitivity of SDS–PMA was assessed by a standard curve. The standard curve was obtained using 10-fold serial dilutions of viable known concentrations of *S. agalactiae*, which were purified to prepare six dilution points ranging from 1 × 10^2^ cfu/ml to 1 × 10^7^ cfu/ml as an external standard. The different bacterial concentrations (log cfu for the reaction) were determined as corresponding Cq values. Linear relationships and slopes for the curves were automatically calculated with Bio-Rad CFX Manager 3.1.

### Detection of *S. agalactiae* in Spiked Milk Using SDS–PMA–qPCR

Ultra-high temperature (UHT) milk (Mengniu, Inner Mongolia, China), confirmed negative for *S. agalactiae* by standard culture method (NY/T 2962-2016, China), was used in the spiking studies. Three groups with different treatments were prepared by inoculation: (i) with 3 × 10^3^ cfu/ml of viable *S. agalactiae*; (ii) with 3 × 10^3^ cfu/ml of viable *S. agalactiae* and 3 × 10^4^ cfu/ml of dead *S. agalactiae*; and (iii) with 3 × 10^4^ cfu/ml of viable *S. agalactiae* and 3 × 10^3^ cfu/ml of dead *S. agalactiae*. Then, the mixtures were treated with or without optimal SDS and PMA.

### Statistical Analysis

All experiments were performed in triplicate. SPSS20.0 software was used to determine statistically significant differences. The limits of detection (LODs) were calculated as the lowest numbers of cells that could be detected by the assays.

## Results

### Inclusivity and Exclusivity of Primers

The inclusivity and exclusivity of the primers used in the qPCR assay were evaluated using target and non-target strains of pathogens (Table 1). The results showed that amplification of *cfb* was positive only in *S. agalactiae* strains, and no amplified signals were observed from DNA of *Cronobacter sakazakii*, *Bacillus cereus*, *Escherichia coli*, *Shigella flexneri*, *Cronobacter muytjensii*, *Salmonella enterica*, *Lactobacillus plantarum*, *Enterococcus faecalis*, *Staphylococcus aureus*, and *Streptococcus salivarius*. The results indicated that the primers were highly specific for *S. agalactiae*, with no cross-reactivity to non-target bacteria.

### Optimization of SDS Treatment

**FIGURE 1:**
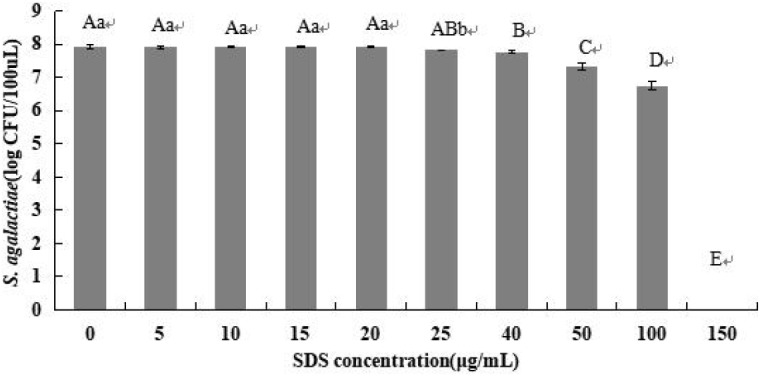
Optimization of SDS concentration. Samples of 8 × 10^7^ cfu/ml of *S. agalactiae* were treated with different SDS concentrations. Error bars indicate standard deviations. Values with the same letter indicate no significant differences (*P* > 0.05); different lower and upper case letters indicate significant differences at *P* < 0.05 and *P* < 0.01, respectively. Color version available online.

The mean bacterial numbers on plates with different concentrations of SDS were determined (Figure 1). For SDS concentrations of 0, 5, 10, 15, 20, 25, 40, 50, 100, and 150 μg/ml, the log cfu values were 7.93, 7.91, 7.92, 7.92, 7.92, 7.82 7.77, 7.32, 6.75, and 0, respectively. There was a sharp decline of log cfu values within 20–25 μg/ml of SDS (*P* < 0.05). However, the *S. agalactiae* colony number decreased significantly when SDS concentration exceeded 40 μg/ml (*P* < 0.01). Hence, an optimum concentration was 20 μg/ml, which did not affect the growth of viable *S. agalactiae*.

### Evaluation of LYZ Concentrations

**FIGURE 2:**
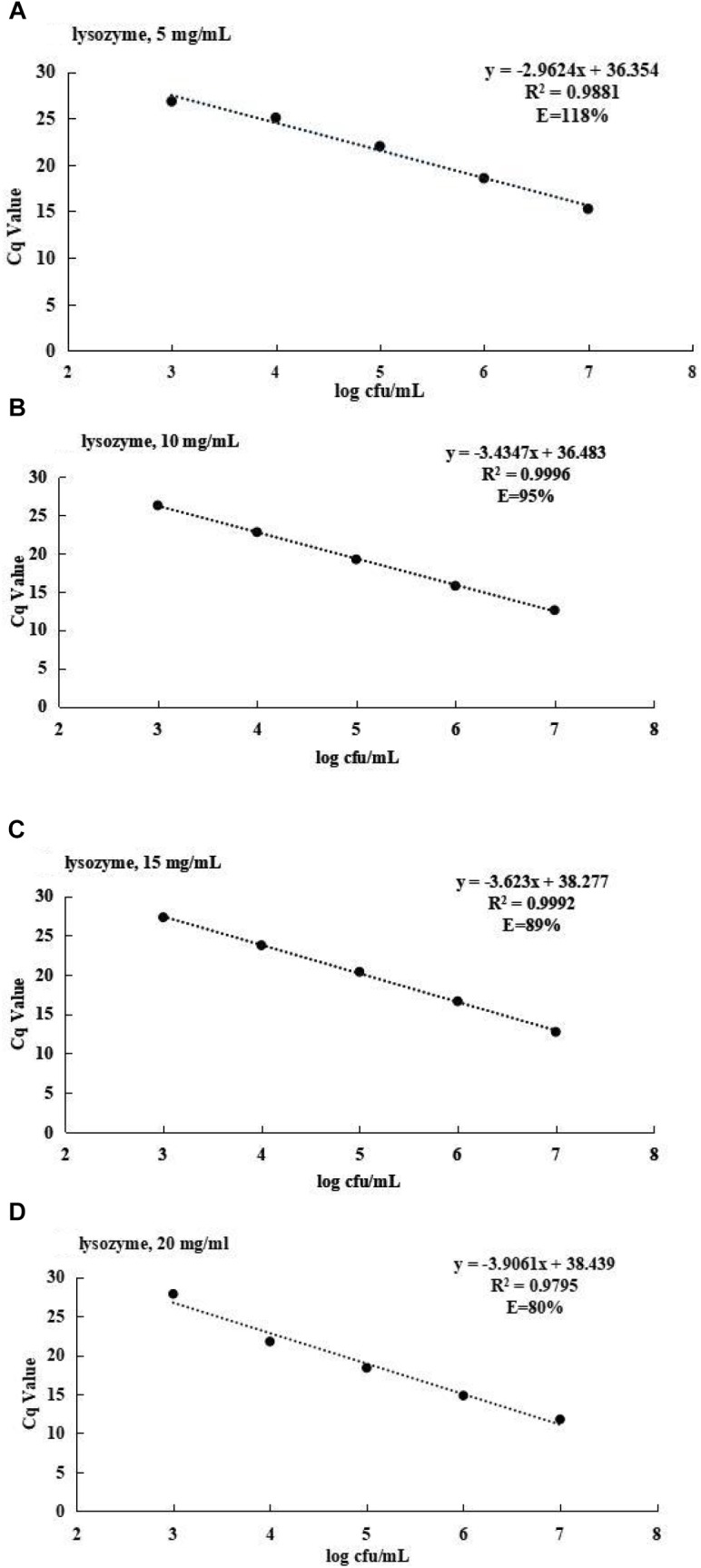
**(A)** The standard curve for *S. agalactiae* at 5 mg/ml of lysozyme. **(B)** The standard curve for *S. agalactiae* at 10 mg/ml of lysozyme. **(C)** The standard curve for *S. agalactiae* at 15 mg/ml of lysozyme. **(D)** The standard curve for *S. agalactiae* at 20 mg/ml of lysozyme.

The LYZ concentration was optimized by standard curve (Figure 2). For LYZ of final concentrations of 5, 10, 15, and 20 mg/ml, the co-efficient of determination (*R*^2^) values were 0.9881, 0.9996, 0.992, and 0.9795, respectively, and corresponding amplification efficiency values were 118, 95, 89, and 80%. Therefore, the optimal LYZ concentration was 10 mg/ml.

### Optimization of PMA Treatment

**FIGURE 3:**
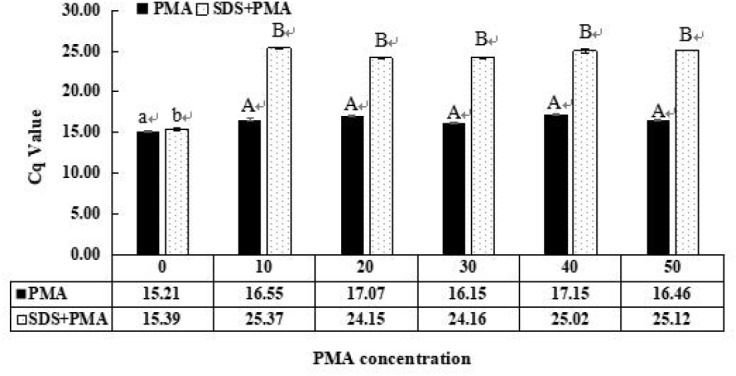
Optimization of propidium monoazide (PMA) concentration that inhibits amplification of dead *S. agalactiae* cells. Samples were treated with 0, 10, 20, 30, 40, and 50 μM PMA. Error bars indicate standard deviations. Values with the same letter indicate no significant difference (*P* > 0.05); different lower and upper case letters indicate significant differences at *P* < 0.05 and *P* < 0.01, respectively. Color version available online.

The different PMA concentrations on dead and viable *S. agalactiae* cells were investigated to determine the optimal PMA treatments. The Cq values with standard deviation are shown in Figure 3. The Cq value with SDS treatment was higher than no treatment (*P* < 0.05); the Cq value of SDS–PMA was significantly higher than only PMA treatment when the mixed bacteria suspension was incubated with different PMA concentrations (*P* < 0.01). The highest Cq value was 23.37, and the corresponding PMA concentration was 10 μM. These results showed that 10 μM PMA was the optimal concentration to bind to DNA from injured or dead cells.

### Sensitivity of SDS–PMA–qPCR Assay

**FIGURE 4:**
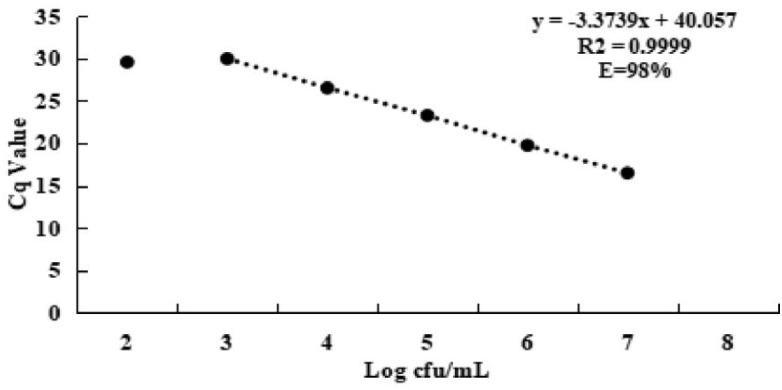
Standard curve for *S. agalactiae* of SDS–PMA–qPCR assay, plotted values represented the mean value and standard deviations obtained from three triplicate tests. Cq = threshold cycle. Color version available online.

A good linear correlation between Cq value and the number of viable *S. agalactiae* cells was obtained from SDS–PMA–qPCR standard curve (Figure 4), with *R*^2^ = 0.999 and slope of −3.3739. The amplification efficiency value (*E*) was 98%, calculated using a formula: *E* = (*e*^−1/*slope*^ − 1) × 100%. The results indicated that the SDS–PMA–qPCR assay was highly linear over the range 10^3^–10^7^ cfu/ml, and LOD of the method was 10^3^ cfu/ml.

### Detection of Viable *S. agalactiae* in Spiked Milk

**FIGURE 5:**
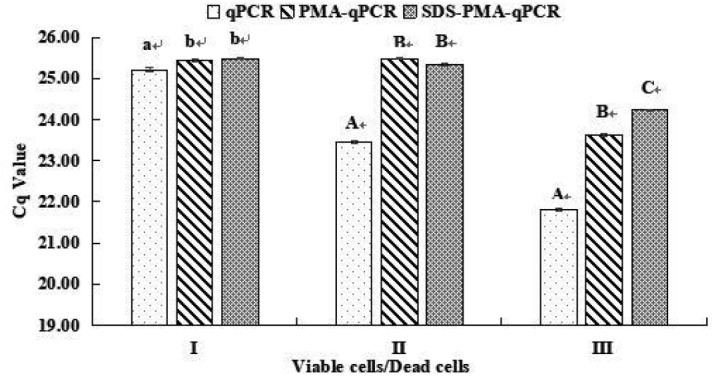
Quantification of viable and viable/dead mix of *S. agalactiae* cells by qPCR, PMA–qPCR, and SDS–PMA–qPCR methods. Milk samples inoculated with **(I)** 3 × 10^3^ cfu/ml of viable cells, **(II)** 3 × 10^3^ cfu/ml of viable cells and 3 × 10^4^ cfu/ml of dead cells, and **(III)** 3 × 10^4^ cfu/ml of viable cells and 3 × 10^3^ cfu/ml of dead cells. All tests were carried out in duplicate. Methods for the preparation of dead cells and mixed viable and dead cells are described in Materials and Methods. Values within the same group (**I**, **II**, and **III**) with different lower (a, b) and upper (A, B, C) case letters are significantly different by Duncan’s multiple range test at *P* < 0.05 and *P* < 0.01, respectively.

Viable *S. agalactiae* in spiked milk were detected by SDS–PMA–qPCR. The levels of *S. agalactiae* cells in spiked milk for the various methods were compared to validate efficiency (Figure 5). The Cq values were 25.48 with SDS and PMA treatment, 25.45 with PMA treatment and 25.21 without SDS and PMA treatment when milk samples were inoculated with 3 × 10^3^ cfu/ml of viable *S. agalactiae* cells with LYZ. The variations in the Cq value can reflect the content of viable *S. agalactiae* in spiked milk (the lower the Cq value, the higher was the *S. agalactiae* content). The *S. agalactiae* levels quantified by PMA–qPCR and SDS–PMA–qPCR were significantly lower than for qPCR (*P* < 0.05, Figure 5I).

When milk samples were inoculated with 3 × 10^3^ cfu/ml of viable *S. agalactiae* cells and 3 × 10^4^ cfu/ml of dead cells, and had LYZ added, the Cq values were 25.35 with SDS–PMA treatment, 25.48 with PMA treatment and 23.46 without SDS and PMA treatment. The *S. agalactiae* levels for PMA–qPCR and SDS–PMA–qPCR were significantly lower than for qPCR (*P* < 0.01, Figure 5II). Moreover, *S. agalactiae* levels of PMA–qPCR and SDS–PMA–qPCR did not significantly differ under this condition. However, when UHT milk had 3 × 10^4^ cfu/ml of viable *S. agalactiae* cells and 3 × 10^3^ cfu/ml of dead cells with LYZ added, the Cq values were 24.23 with SDS–PMA treatment, 23.63 with PMA treatment and 21.80 without SDS and PMA treatment. There were significant differences between qPCR, PMA–qPCR and SDS–PMA–qPCR (*P* < 0.01, Figure 5III).

## Discussion

*Streptococcus agalactiae* is the main causal pathogen of bovine mastitis, and has a substantial impact on production quality of milk and possessing great hazards to human health. However, it is difficult to detect the viable but nonculturable *S. agalactiae* cells in milk using conventional microbiological methods. Consequently, due to specificity and sensitivity, some studies have developed conventional PCR techniques to detect *S. agalactiae* in this sample (Meiri-Bendek et al., 2002; Duarte et al., 2016). However, the presence of dead cells contributing DNA to the sample, limits the use of PCR for microbiological monitoring of food samples (Wang et al., 2014). Many studies demonstrated the use of PMA–qPCR for detection of pathogenic microorganisms in milk (Wang et al., 2015). Previous researchers successfully used PMA–PCR to detect viable target bacteria in food products including of *Escherichia coli*, *Salmonella* spp., *Enterobacter sakazakii*, *Bacillus cereus*, *Staphylococcus aureus*, *Lactobacillus paracasei*, *L*. *monocytogenes*, *Campylobacter* spp., and *Vibrio parahaemolyticus* (Cawthorn and Witthuhn, 2008; Liu et al., 2012; Xiao et al., 2015; Zhang et al., 2015; Zeng et al., 2016; Takahashi et al., 2017; Scariot et al., 2018). However, using PMA–qPCR to distinguish between viable and dead *S. agalactiae* cells in pasteurized milk has not yet been reported.

In order to achieve high detection specificity and simplify the amplification system, it is critical to select specific target genes and design qPCR primers. In this study, a primer pair targeting *cfb* was chosen to design a qPCR assay for detection of *S. agalactiae* since it had been confirmed to be the conservative gene (El Aila et al., 2011; Kaczorek et al., 2017). In this study, only the target bacterial strains were amplified with the expected size of fragment, and non-target strains were PCR negative (Table 1). This indicated that the primers were highly specific and sensitive for *S. agalactiae*. Thus, the SDS–PMA–qPCR assay showed appropriate inclusivity and exclusivity, and it was reliable in detecting *S. agalactiae*.

Research showed that PMA combined with qPCR could penetrate the compromised cell membranes of dead *S. agalactiae* and eliminate false-positive results – our study confirmed this point. The effective PMA concentration can reduce the performance on viable and dead cell mixtures containing high densities of dead cells (Pan and Breidt, 2007; Contreras et al., 2011; Elizaquível et al., 2014). It has been suggested that the PMA concentration be optimized for different microorganisms to generate reliable results (Fittipaldi et al., 2012). The 10 μM PMA concentration was chosen as optimal to inhibit amplification of dead *S. agalactiae* (Figure 3). Some studies showed that 50 μM PMA significantly inhibited the detection of dead *Staphylococcus aureus* and *Salmonella* spp. in food (Nocker et al., 2006; Kobayashi et al., 2009; Li and Chen, 2013). These findings are not consistent with our results possibly because the degree of penetration of PMA in dead bacteria is related to the type of bacteria. However, combined with the previous reports, the additional PMA treatment method was still insufficient to accurately determine viable cells (Zhou et al., 2016).

To solve this issue, SDS was applied to improve the permeability of dead cells to PMA. The 20 μg/ml SDS concentration used in our study was lower than in previous studies. Takahashi et al. (2017) considered 250 μg/ml as the optimum to detect viable *E. coli*, and Dong et al. (2018) found that 100 μg/ml was optimum for *Staphylococcus aureus*. The cell membrane integrity of different strains differs, and so there is a difference in ability of SDS to combine with dead cells, and this may explain the great difference in SDS concentrations.

Use of LYZ is universal for extraction of DNA from various microorganisms (Fakharany and Hassan, 2016). In an early paper by Boström et al. (2004), LYZ treatment significantly influenced DNA extraction efficiency. Our study verified that LYZ could crack cell membranes and improve effectiveness of DNA extraction, with an optimal concentration of 10 mg/ml.

The reliability of the SDS–PMA–qPCR assay was validated by its application to UHT milk. When milk samples were inoculated with different amounts of dead bacteria, the SDS helped PMA to bind with dead bacteria DNA and so only viable bacteria remaining in milk were detected. This suggests that SDS–PMA–qPCR is suitable for the selective detection and quantification of viable *S. agalactiae* cells in milk. The SDS–PMA–qPCR combined with LYZ resulted in detection down to 3 × 10^3^ cfu/ml in the spiked milk matrix in our study. To our knowledge, the LOD of *S. agalactiae* using SDS–PMA–qPCR has not been previously reported. Zhang et al. (2015) found that the LOD of PMA–qPCR was 1.55 × 10^2^ cfu/ml in pure culture and 3 × 10^2^ cfu/ml in milk powder, and had greater sensitivity than conventional PCR with 1.5 × 10^4^ cfu/ml. The LOD for sodium deoxycholate–PMA–qPCR with immunomagnetic separation for *E. coli* O157:H7 in spiked milk matrix was 10^2^ cfu/ml (Wang and Levin, 2006). The reason for this difference might be the different proportions of viable target bacteria, as well as different concentrations of PMA and types of surfactants. We will continue to optimize the SDS–PMA–qPCR method to detect lower microbial counts from samples in future studies.

When milk samples were inoculated with 3 × 10^3^ cfu/ml of viable cells only, the level of *S. agalactiae* significantly differed between PMA–qPCR, SDS–PMA–qPCR, and qPCR (*P* < 0.05). This might be due to the presence of some injured cells in mixtures of viable bacteria under the pressure of light – the light exposure step is necessary to activate the PMA bound to DNA of dead cells and inactivate the excess PMA that has not entered cells (Fittipaldi et al., 2011). When milk samples were inoculated with 3 × 10^4^ cfu/ml of viable *S. agalactiae* cells and 3 × 10^3^ cfu/ml of dead cells or 3 × 10^3^ cfu/ml of viable cells and 3 × 10^4^ cfu/ml of dead cells, the difference in results for PMA–qPCR compared with SDS–PMA–qPCR indicated that the presence of dead cell debris with an intact outer membrane was more efficiently affected by the SDS–PMA combination than by PMA alone. Previous studies showed that DNA of dead cells of various pathogens in milk, meat homogenates or water samples can be inactivated for PCR by treatment with PMA or SDS–PMA (Tomás et al., 2009; Gensberger et al., 2014; El-Aziz et al., 2018). Compared with these reports, the PMA–qPCR assay with SDS developed in the present study was faster and had higher sensitivity than previously reported methods. The low PMA concentration in our study might also be an important reason for the effective passivation of dead bacteria by the SDS–PMA combination. We found that the higher proportion of viable cells, the closer were the quantitative results of SDS–PMA–qPCR to the viable cells in spiked bacterial samples. This indicated that the PMA–SDS combination successfully distinguished viable and dead *S. agalactiae* by inhibiting amplification of DNA of dead bacteria. In the quantitative detection of *S. agalactiae* by SDS–PMA–qPCR, the viable bacteria in the milk sample were accurately reflected and dead bacteria in the background were eliminated. As far as we know, this is the first time that viable *S. agalactiae* have been detected in UHT milk using SDS–PMA–qPCR.

## Conclusion

We developed a specific, sensitive, and accurate SDS–PMA–qPCR assay in combination with LYZ for detection of viable *S. agalactiae* in milk without the interference of false-positives and -negatives. The LOD was 3 × 10^3^ cfu/ml for SDS–PMA–qPCR used to detect viable *S. agalactiae* in milk matrix. The accurate and rapid SDS–PMA–qPCR combination we developed holds promise for quantitative detection and monitoring of *S. agalactiae* contamination in milk in field conditions.

## Author Contributions

YZ, HC, HL, and CW conceived and supervised the study. YZ, HL, LM, NZ, and JW designed the experiments. YZ, JC, and LD performed the experiments. YZ, HL, LM, LD, and JC analyzed the data. YZ and HL revised the manuscript. YZ wrote the manuscript.

## Conflict of Interest Statement

The authors declare that the research was conducted in the absence of any commercial or financial relationships that could be construed as a potential conflict of interest.
